# A Systematic Review of the Evidence Supporting Mobile‐ and Internet‐Based Psychological Interventions For Self‐Harm

**DOI:** 10.1111/sltb.12583

**Published:** 2019-08-26

**Authors:** Usman Arshad, Farhat‐ul‐Ain, Jessica Gauntlett, Nusrat Husain, Nasim Chaudhry, Peter James Taylor

**Affiliations:** ^1^ Pakistan Institute of Living & Learning Karachi Pakistan; ^2^ Division of Psychology & Mental Health Manchester Academic Health Sciences Centre School of Health Sciences University of Manchester Manchester UK

## Abstract

**Objectives:**

Internet‐ and mobile phone‐based psychological interventions have the potential to overcome many of the barriers associated with accessing traditional face‐to‐face therapy. Self‐injurious thoughts and behaviors (STB) are prevalent global health problems that may benefit from Internet‐ and mobile‐based interventions. We provide a systematic review and meta‐analysis of studies evaluating mobile‐ and Internet‐based interventions for STB, including nonsuicidal self‐injury (NSSI).

**Methods:**

Online databases (PsycINFO, Web of Science, Medline) were searched up to March 2019 for single‐arm and controlled trials of Internet‐ or mobile‐based interventions for STB. The potential for bias was assessed using the Cochrane risk of bias tool.

**Results:**

Twenty‐two eligible trials were identified. The research was limited by a lack of controlled designs and small samples. Evidence supports the acceptability of interventions. There is preliminary evidence that these interventions are associated with a decline in STB. A meta‐analysis suggested a positive treatment effect on suicidal ideation when compared to treatment as usual, but not when trials with active controls were also considered.

**Conclusions:**

Overall, Internet‐ and mobile‐based interventions show promise and further controlled trials are warranted, focusing on behavioral outcomes (NSSI, suicidal behavior).

This review was preregistered with PROSPERO (CRD42017074065).

Suicide is a serious global public health issue ranked among the leading causes of death in many countries (World Health Organisation, 2014). Self‐injurious thoughts and behavior (STB) are associated with an increased risk of suicide (Grandclerc, De Labrouhe, Spodenkiewicz, Lachal, & Moro, 2016; Hawton, Bergen, et al., 2015; Ribeiro et al., 2016; Scott, Pilkonis, Hipwell, Keenan, & Stepp, 2015). Even where STB does not lead to suicide, it is associated with a reduced life span, and greater physical and mental health difficulties (Bergen et al., 2012; Goldman‐Mellor et al., 2014; Hawton, Saunders, & O'Connor, 2012). STB encompasses self‐injurious behaviors (SB), which can be divided into those behaviors with suicidal intent (i.e., suicide attempts) and nonsuicidal self‐injury (NSSI), and self‐injurious thoughts, which include suicidal ideation (Ribeiro et al., 2016; Silverman, Berman, Sanddal, O'Carroll, & Joiner, 2007).

There is evidence that psychological and social interventions can be effective in preventing STB (Hawton, Witt, et al., 2015; Hawton et al., 2017; Hetrick, Robinson, Spittal, & Carter, 2016; Turner, Austin, & Chapman, 2014). There is preliminary support for the efficacy of a number of psychological therapies for people who struggle with STB, including Cognitive Behavioral Therapy (CBT), Emotion‐Regulation Group Therapy (ERGT), and Dialectical Behavioral Therapy (DBT; Hawton, Witt, et al., 2015; Hawton et al., 2017; Hetrick et al., 2016; Turner et al., 2014). However, these therapies are typically costly in terms of resources, the necessity of trained therapists, and the duration of therapy typically required. While there is also preliminary evidence for some brief psychological therapies for STB (Guthrie et al., 2001; Tapolaa, Lappalainen, & Wahlström, 2010), a limitation of the suicide prevention literature is that the majority of interventions rely on face‐to‐face contact (delivered at a group, family, community, or individual level). These interventions may therefore not be accessible for many individuals, due to geographical (e.g., living in rural setting with limited mental health resources, travel distance to appointments), social (e.g., barriers related to stigma), organizational (e.g., waiting times and service availability), or even financial reasons (e.g., in healthcare contexts where clients must pay for their treatment; Department of Health, 2017; Institute of Medicine, 2002; Leigh & Flatt, 2015; Poppleton & Gire, 2017). Approximately seventy‐eight percent of suicides occur in low‐ and middle‐income countries (World Health Organisation, 2018). These locations are therefore an important focus for STB and suicide prevention, but challenges related to access to treatment are also often exacerbated in these contexts. Challenges facing low‐ and middle‐income countries include fewer available mental health practitioners relative to the population size and a lack of mental health services and staff in more rural areas (Rathod et al., 2017). Internet‐ or mobile‐based interventions may be one avenue for overcoming these challenges (Lai, Maniam, Chan, & Ravindran, 2014; Leigh & Flatt, 2015; Poppleton & Gire, 2017). Stakeholders have identified improved access to treatment as a key benefit of Internet‐ or smart phone‐mediated approaches to helping suicidal individuals (Ward‐Ciesielski, Peros, Conigliaro, & Gilmore, 2018). Such interventions have the potential to be delivered remotely to provide an alternative way of helping those at risk of STB where access to face‐to‐face interventions is limited. The present review focuses on the evidence base for these interventions.

mHealth or mobile health, referring to health interventions that are delivered or supported via remote devices such as mobile phones, wearable technology, or personal digital assistants (PDAs), is increasingly being considered as a basis for mental health intervention (Bucci et al., 2015; Sort, 2017). Such interventions can be considered part of the broader body of Internet‐based interventions, or eHealth (e.g., interventions delivered via Web sites and browser‐based applications; Christensen, Batterham, & O'Dea, 2014). There is evidence that Web‐ and mobile‐based interventions may be effective for common mental health difficulties including depression and anxiety (Firth et al., 2017; Josephine, Josefine, Philipp, David, & Harald, 2017; Richards, Richardson, Timulak, & McElvaney, 2015; Stratton et al., 2017). A previous review of eHealth interventions for suicide prevention noted that they may be beneficial, but were likely to be more effective where specifically focused on suicide rather than related problems like depression (Christensen et al., 2014). A large number of mHealth interventions aimed at STB prevention have been developed (Larsen, Nicholas, & Christensen, 2016). However, research regarding the acceptability, feasibility, and efficacy of such interventions appears scarcer (Christensen et al., 2014; Melia et al., 2018; Perry, Werner‐Seidler, Calear, & Christensen, 2016).

Despite the potential benefits of Internet‐ and mobile‐based interventions for STB, there are also reasons for caution. The relationship between client and therapist appears to be a key factor in influencing the outcome of therapy (Blake, Larkin, & Taylor, 2019; Goldsmith, Lewis, Dunn, & Bentall, 2015; Michel, 2016). It has been suggested that Web‐ and mobile‐based interventions may have a more limited capacity to facilitate a therapeutic relationship due to the lack of a face‐to‐face contact (Perle, Langsam, & Nierenberg, 2011). There is preliminary evidence that a positive working alliance (one aspect of the therapeutic relationship) can develop with computer‐based interventions in individuals with mild‐to‐moderate mental health difficulties (Clarke et al., 2016). However, it remains unclear whether Web‐ or mobile‐based interventions could provide an effective alternative in more complex contexts, including those at risk of STB. Clinicians have concerns about the use of such approaches with clients at risk of suicide, including the lack of a thorough assessment, and constraints on being able to respond to elevated risk (Gilmore & Ward‐Ciesielski, 2017). A further potential issue is the reduced capacity for the collaborative development of understanding. Many therapeutic approaches rely on the development of a personalized understanding (or formulation) of a client's difficulties, including how problems have developed and what has kept them going, which can guide the therapy (e.g., Tarrier & Johnson, 2016; Taylor, Gianfrancesco, & Fisher, 2019). The interaction with a therapist is key in guiding this development, and it may not be possible to replicate this process within mobile‐ or Web‐based interventions. Consequently, questions can be raised about both the acceptability and efficacy of mobile‐ and Web‐based interventions for individuals who struggle with STB.

Given the increasing number of studies evaluating mobile‐ and Web‐based interventions for STB prevention, it is timely and important to evaluate the extant evidence regarding the acceptability, feasibility, and efficacy of mobile‐ and Web‐based intervention for STB (including suicidal behavior) and suicidal ideation. The current review aimed to expand on previous reviews in this area by focusing specifically on interventions aimed at STB (rather than associated difficulties like depression), providing a synthesis of acceptability and feasibility results alongside efficacy, inclusion of a number of more recent trials in this area not covered by previous reviews, preregistration of the review protocol, and the use of meta‐analysis incorporating a number of new trials. The aim of this study was to systematically review the current evidence for (1) the efficacy of Web‐ and mobile‐based interventions in reducing STB and suicidal ideation in adults and young people at risk of STB; (2) the acceptability of these interventions, operationalized in terms of participant feedback on the experience of using the intervention (e.g., Proctor et al., 2011); and (3) the feasibility of these interventions, operationalized in terms of rates of engagement with the intervention (Proctor et al., 2011). We considered both active control interventions and treatment as usual. Regarding acceptability and feasibility, we recognize that these concepts can overlap with the “usability” of software (i.e., on a practical level how easy and intuitive the software is to use). For example, if software is difficult or confusing to use, participants are more likely to stop using it.

## Method

### Preregistration

A protocol for this review was preregistered with PROSPERO (CRD42017074065). The PRISMA reporting guidelines were adhered to in this review (Moher, Liberati, Tetzlaff, Altman, & The, 2009). Departures from protocol are listed in Appendix S1.

### Search Strategy

Electronic databases (PsycINFO, Web of Science, and MEDLINE) were searched for eligible studies. The original search, specified in the protocol, was conducted from the earliest date up to January 2019. The details of this search are available in Appendix S2. Based on reviewer feedback, we adjusted our search terms and repeated the search from earliest date available up to March 2019. For this revised search, the following subject terms and Boolean operators were used: (web OR online OR internet OR “mobile device” OR “cell* phone” OR “smartphone” OR “phone app” OR “mobile app” OR “cell* app” OR “tablet computer” OR iPad OR iPhone OR Samsung OR android OR “windows phone”) AND (self‐harm” OR “self‐injury” OR DSH OR NSSI OR “self‐burn*” OR “self‐mutilation” OR “self‐cutting” OR suicid* OR overdose*) AND (therapy OR intervention OR treatment OR training). The asterisks indicate wildcard operators. These terms were informed by those used in previous reviews in this area (e.g., Witt et al., 2017). The original pre‐protocol search did not identify any eligible studies that were not also picked up in the revised search. Because the literature on mobile and Internet‐based applications also overlaps the fields of software design and development, a further search, from earliest date up to March 2019, was conducted on the Association of Computing Machinery digital library (ACMD) using the terms: self‐harm” OR “self‐injury” OR DSH OR NSSI OR “self‐burn*” OR “self‐mutilation” OR “self‐cutting” OR suicid* Or overdose*.

These online literature searches were supplemented by (1) checking for any additional potentially eligible papers cited by included articles; (2) contacting all corresponding authors of included articles inquiring whether they have any other studies (published or not) that might be eligible for the review); and (3) checking reference lists of relevant reviews (Christensen et al., 2014; Lai et al., 2014; Perry et al., 2016; Witt et al., 2017).

The data screening was done in two steps. Firstly, titles and abstracts were screened and studies not fulfilling the inclusion criteria were excluded. Where it was uncertain if studies met inclusion criteria, they were retained for the next stage of screening. Secondly, full‐text articles were screened out on the basis of inclusion/exclusion criteria. Both stages of screening were completed in parallel by two independent reviewers (active researchers with masters or doctoral level qualifications in psychology) and discrepancies resolved through discussion.

### Inclusion and Exclusion Criteria

Both published literature and gray literature were included. Trials were eligible for inclusion provided that they met the following criteria: (1) Intervention studies include single‐arm trials, case series, and randomized controlled trials; (2) participants characterized by experiences of STB; (3) the intervention is an Internet‐ or mobile phone‐delivered psychological intervention broadly focused on STB prevention. We excluded a number of studies where the intervention was directed more broadly at depression and the sample was not characterized by experiences of STB (e.g., Mewton & Andrews, 2015). This was important given evidence that interventions directed primarily at other difficulties where STB is a secondary outcome tend to be less effective (Christensen et al., 2014; Tarrier, Taylor, & Gooding, 2008). Developments in mHealth and eHealth focused on assessment or screening for STB risk were also not included. Studies were excluded if (1) exclusively qualitative methodology was used (studies with mixed method were considered); (2) non‐English studies were excluded, only if full‐text English translation was not available.

### Potential for Bias

We assessed potential for bias for each included study using the Cochrane Collaboration Risk of Bias tool (Higgins et al., 2011). Each study was rated as high, unclear, or low potential of bias with respect to the following: adequacy of the random sequence generation procedure, adequacy of allocation concealment, presence of participant and clinical personnel blinding, presence of outcome assessor blinding, presence of incomplete outcome data, presence of selective outcome reporting, and presence of any other bias. Potential for bias ratings were made independently by at least two reviewers (UA, F‐UA, PJT; researchers with master's or doctoral level qualifications in psychology). Discrepancies were resolved through discussion.

### Data Extraction

Quantitative information was extracted independently by at least two reviewers (UA, F‐UA, or PJT) using a data extraction spreadsheet. Extracted information included study characteristics, study design, sample characteristics (for both intervention and control group), intervention characteristics, and results. Any disagreements were resolved after discussion among the team members. Study authors were contacted to provide additional information where data were missing or unclear. The primary outcomes were STB (including suicide attempts), feasibility, and acceptability. Acceptability was based upon participant reported experiences of using the intervention, specifically how helpful or useful the intervention was, or the perceived likelihood of using it again in the future. Feasibility was based upon intervention engagement or usage rates (e.g., number of times accessing a mobile phone app during the study period). Suicidal ideation was also included as a secondary outcome.

### Meta‐Analysis

Meta‐analysis was undertaken where five or more trials were identified with data on a specific outcome (STBs, see protocol). Meta‐analysis was only undertaken for RCTs due to the problems with aggregating effects from single‐arm studies (Cuijpers, Weitz, Cristea, & Twisk, 2016). Following screening, there were only enough trials to undertake meta‐analysis for suicidal ideation. Suicidal ideation was treated as a continuous scale representing severity or frequency of ideation. Hedge's *g* was used to represent these effect sizes. Due to the variety of different assessment tools used, the unstandardized mean difference could not be compared across studies. Endpoint data were used to compare trial arms but a sensitivity analysis looking at mean change score where available was also undertaken. The analyses focused on the time‐point closest to end of treatment. For one trial, the 6‐month follow‐up data were used because following this point both arms of the trial received the mobile‐based intervention preventing a comparison (Marasinghe, Edirippulige, Kavanagh, Smith, & Jiffry, 2012). Where data were unavailable, this was requested from authors. A random‐effects model, using the DerSimonian and Laird (1986) inverse variance estimator, was adopted as heterogeneity in treatment model and outcome assessment was expected. Inconsistency was assessed via the *I* ^2^ statistic (Higgins & Thompson, 2002; Higgins, Thompson, Deeks, & Altman, 2003). Analyses were undertaken in STATA 14 (StataCorp, 2015).

## Results

### Study Characteristics

**Figure 1:**
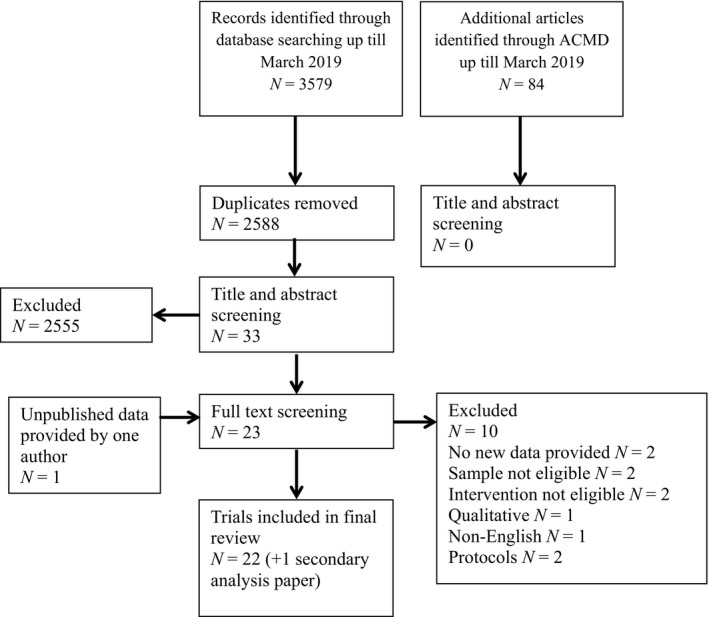
Flowchart of literature screening.

This systematic review identified 22 eligible trials in total (see Figure 1 for flowchart of screening process), one of which was unpublished trial data provided by the author (Eylem, 2019; Eylem, van Straten, Bhui, & Kerkhof, 2015). Study characteristics are summarized in Table 1. Twelve were randomized controlled trials (RCTs), nine were single‐arm studies, and one used a cross‐over counterbalanced controlled design. Twenty‐one studies were from higher income countries including the USA, UK, China, Denmark, Australia, Sweden, Netherland, Japan, Belgium, and France. Only one study was from a middle or lower income country, namely Sri Lanka (Marasinghe et al., 2012).

**Table 1:** Study Characteristics

Author(s) year, Country	Study design	Sample characteristics	Follow‐up period	Intervention	Control	Measures of self‐harm and suicidal ideation	Key results
Berrouiguet et al. (2014), France	Single arm	*N* = 18 Adults with a suicide attempt history; Age *M* = 37.9 years, *SD* = 8.6; Female *N* = 15 (93%)	2 months	Text Messages. Personalized supportive text messages following discharge. Four messages were sent over 30 days, validating experiences and encouraging help‐seeking if needed	NA	Psychiatrists’ report	Overall positive response from participants concerning acceptability of intervention
Bjureberg et al. (2018), Sweden	Single arm	*N* = 25 Adolescents aged 13 to 17 years meeting criteria for NSSI disorder; Age *M* = 15.7 years, *SD* = 1.3; Female *N* = 19 (76%)	6 months	Internet Web site. Eleven module individual emotion‐regulation therapy. Includes individualized crisis plan. Also parent version with nine modules. Delivered over 12 weeks	N/A	Deliberate self‐harm inventory (Bjärehed & Lundh, 2008)	Significant reduction in NSSI frequency (pretreatment to 6 months of follow‐up, *d *=* *1.36, 95% CI: 1.12, 1.63) Good average completion rates of intervention by adolescents (*M *=* *9.7 modules completed out of 11)
Bush et al. (2015), USA	Cross‐over trial	*N* = 18 Adult veterans diagnosed with posttraumatic stress disorder, depression, bipolar disorder, borderline personality disorder or mood disorder, deemed at high risk of SB; Age *M* = 41.4 years, *SD* = 8.6; Female *N* = 10 (56%)	8 weeks	Mobile Phone Application. Suicide prevention application providing a virtual “hope box,” signposting, and coping skills. Used over 6–8 weeks	NA	NA	Overall positive response from participants concerning acceptability of intervention
Bush et al. (2017), USA	RCT	Adult veterans with recent suicidal ideation; Treatment group: *N* = 58; Age *M* = 46.5 years, *SD* = 13.8; Female *N* = 22 (38%) Control group: *N* = 60; Age *M* = 48.7 years, *SD* = 14.3; Female *N* = 15 (25%)	12 weeks	Mobile Phone Application. Suicide prevention application providing a virtual “hope box,” signposting, and coping skills. Used over 12 weeks	Treatment as usual supplemented with printed material about coping with suicidal feelings	First five‐items of the Beck Scale for Suicidal Ideation (BSS; Beck, Kovacs, & Weissman, 1979)	No significant differences (*p *>* *.05) in treatment groups for suicidal ideation at any follow‐up point
Chen et al. (2010), China	Single‐arm	*N* = 15 Adults presenting at hospital due to a suicide attempt; 60% aged < 35 years; Female *N* = 12 (80%)	4 weeks	Text messages. Supportive text messages delivered to mobile phone or smartphone delivered over 4 weeks encouraging help‐seeking	NA	NA	The majority of participants (80%) were positive about the value of the text messages and wished to continue to receive them
Eylem et al. (2019), Netherlands	RCT	*N* = 18 Turkish adults living in the Netherlands with suicidal ideation; Age *M* = 33.5, *SD* = 8.4; Female *N* = 13 (72%)	3 months	Internet Web site. Eight online modules drawing on CBT and DBT principles, Delivered across 6 weeks	Waiting list	BSS	NA
Franklin et al. (2016), USA	RCT	Adults with two or more episodes of self‐cutting in past month; Sample 1: *N* = 114; Age *M* = 23.0 years, *SD* = 5.5; Female *N* = 92 (81%) Sample 2: *N* = 131; Age *M* = 22.9 years, *SD* = 5.0; Female *N* = 97 (74%) Sample 3: *N* = 163; Age *M* = 24.5 years, *SD* = 6.6; Female *N* = 96 (59%)	2 months	Mobile Phone/Computer Application. Therapeutic Evaluative Conditioning (TEC). Game‐like intervention based on behavioral conditioning pairing self‐harm related stimuli with aversive stimuli. Used over 1 month	Nonactive control version of TEC	The Self‐Injurious Thoughts and Behaviors Interview (SITBI; Nock, Holmberg, Photos, & Michel, 2007)	Fewer NSSI episodes in treatment group at 1 month (Sample 1: *M *=* *9.33, *SE *=* *2.08; Sample 2: *M *=* *22.25, *SE *=* *4.71; Sample 3: *M *=* *6.84, *SE *=* *1.37) compared to control (Sample 1: *M *=* *21.52, *SE *=* *5.34; Sample 2: *M *=* *23.19, *SE *=* *6.24; Sample 3: *M *=* *6.71, *SE *=* *1.98). Difference significant (*p *<* *.05) in two of the three samples. Treatment effects not maintained at 2 month follow‐up
Hetrick et al. (2016), Australia	RCT	*N* = 50 High school students in contact with well‐being staff members with suicidal ideation in past 4 weeks; Age *M* = 14.7 years, *SD* = 1.4; Female *N* = 41 (82%)	22 weeks	Internet Web site. CBT‐based intervention delivered across eight modules over 10 weeks, focused on suicidal thinking and behavior	Treatment as usual	Suicidal Ideation Questionnaire (Reynolds, 1987); Nonvalidated measure of suicidal behavior	Decrease in suicidal ideation from baseline to week 10 greater in treatment group (*M* change = –37.3, *SD *=* *39.1) compared to control (*M* change = −31.6, *SD *=* *42.8), but difference not significant (*p *=* *0.59). Also no significant treatment effect at 22 weeks
Hooley et al. (2018), USA	RCT	*N* = 144 Adults with two or more episodes of NSSI in the past month; Age *M* = 25.63 years, *SD* = 5.83; Female *N* = 123 (85%)	16 weeks	Internet Web site. Autobiographical Self‐Enhancement Training (ASET). Involves writing exercises focused on identifying and focusing on positive personal characteristics. Delivered over 4 weeks	Expressive writing; Journaling	SITBI	No significant differences (*p *>* *.05) between groups for the number of NSSI episodes at end of treatment, 1 month or 2 month follow‐up
Kennard et al. (2018), USA	RCT	*N* = 66 Adolescents hospitalized due to suicidal ideation or suicide attempt; Age *M* = 15.1 years, *SD* = 1.5; Female *N* = 59 (89%)	24 weeks	Face‐to‐face therapy supplemented with mobile phone application. The mobile phone application delivers CBT‐ and DBT‐informed skills training and safety planning interventions	Treatment as usual	Suicidal Ideation Questionnaire‐Junior version (SIQ‐J; Reynolds, 1987); Columbia Suicide Severity Rating Scale (CSSRS: Posner et al., 2011)	No significant association (*p *>* *.05) between app use frequency and changes over time in suicidal ideation or behavior
Kodama et al. (2016), Japan	Single arm	*N* = 30 psychiatric outpatients; Age *M* = 38.4 years, *SD* = 11.4; Female *N* = 15 (50%)	6 months	Text messages. Supportive text messages delivered to mobile phone or smartphone delivered over 6 months	NA	Psychiatrists’ report	Significant effect of time (*p *<* *.05) whereby proportion who reported SB decline from 28% to 7% Significant effect of time (*p *<* *.05) whereby intensity of suicidal ideation declined from *M *=* *2.00, *SD *=* *1.18, to *M *=* *0.83, *SD *=* *1.00
Marasinghe et al. (2012), Sri Lanka	RCT	*N* = 68 Patients admitted to hospital after attempting self‐harm, and displaying significant suicidal intent; Intervention group: Age *M* = 34.0, *SD* = 14.0–17.0; Female *N* = 17 (50%) Control group: Age *M* = 29.0–31.0, *SD* = 16.0–17.0; Female *N* = 17 (50%)	6 and 12 months	Text messages. Text message reminders concerning coping skills, use of support and signposting. These follow face‐to‐face and telephone intervention. Delivered over 12 months	Wait‐list/usual care	BSS	Significant time by group effect (*p *<* *.05) whereby suicidal ideation declined more over 12 months for the treatment group
McManama O'Brien et al. (2016), USA	Single arm	*N* = 20 Adolescent mental health outpatients; Age *M* = 15.7 years, *SD* = 1.6; Female *N* = 16 (80%)	NR	Mobile Phone Application. Suicide prevention application providing signposting, and coping skills. Modules are also available to help support parents	NA	NA	Overall positive response from participants concerning acceptability of intervention
O'Toole et al. (2019), Denmark	RCT	Adult psychiatric outpatients with current suicidal ideation. Treatment group: *N* = 60; Age *M* = 28.1 years, *SD* = 9.2; Female *N* = 24 (40%) Control group: *N* = 69; Age *M* = 29.3 years, *SD* = 9.7; Female *N* = 30 (44%)	4 months	Mobile phone application. Application includes psychoeducation, self‐assessment, and safety planning components as well as library of self‐help exercises	Treatment as usual (including psychotherapy)	NA	The majority of participants made use of the app (83%) but ratings regarding the role of the app in overall treatment were neutral
Pauwels et al. (2017), Belgium	Single arm	*N* = 21 adults with some degree of suicidal ideation; Age *M* = 30.0 years; Female *N* = 16 (76%)	1 week	Mobile Phone Application. Series of components to help during a suicidal crisis including coping strategies (based upon CBT principles), safety and crisis planning, support in accessing social network. Delivered over 1 week	NA	BSS	Mixed evidence of acceptability. Seventy percent of participants indicated they would use the app in daily life but 20% also said it did not help with suicidal thoughts
Rizvi et al. (2016), USA	Single arm	*N* = 16 Adults diagnosed with borderline personality disorder with a recent history of repeated NSSI and/or suicide attempt; Age *M* = 27.5 years, *SD* = 7.1; Female *N* = 12 (75%)	9 months	Mobile Phone Application. DBT skills training and coaching delivered across four modules. Taking place alongside face‐to‐face therapy. App available for 9 months	NA	SITBI	App usage was significantly associated (*p *<* *.05) with a decline in NSSI, explaining 26% of within‐person variance in NSSI. App usage was not significantly (*p *>* *.05) associated with changes in suicide attempts
Robinson et al. (2016), Australia	Single arm	*N* = 34 High school students in contact with well‐being staff members with suicidal ideation in past month; Age *M* = 15.6 years; Female *N* = 28 (88%)	8 weeks	Internet Web site. CBT‐based intervention delivered across eight modules focused on suicidal thinking and behavior (same intervention used by Hetrick et al., 2017)	NA	SIQ‐J for year 8 and 9 students and the Adult Suicidal Ideation Questionnaire for older students (Osman et al., 1999)	There was a significant reduction (*p *<* *.05) in suicidal ideation from baseline to posttreatment, *d *=* *0.66, *p *<* *.01
Stallard et al. (2018), UK	Single arm	*N* = 44 Young people aged 12 to 17 years with a history of self‐harm; Age *M* = 16.0 years, *SD* = 1.4; Female *N* = 40 (91%)	12 weeks	Mobile Phone Application. Toolbox of strategies derived from CBT and DBT principles. Delivered over 12 weeks	NA	Nonvalidated self‐report measure of self‐harm	The number of participants reporting SB in the past 4 weeks declined from 79% at baseline to 67% posttreatment
Tighe et al. (2017), Australia	RCT	*N* = 61 Adults with moderate or greater depression and suicidal thoughts in last 2 week; Age *M* = 26.3 years, *SD* = 8.1; Female *n* = 39 (64%)	6 weeks	Mobile Phone Application. Suicide prevention skills training intervention including mindfulness, self‐soothing and acceptance‐based techniques alongside emergency contact signposting, delivered across three modules over 6 weeks	Wait‐list/usual care	The depressive symptom inventory‐suicidality subscale (Metalsky & Joiner, 1997)	No significant time by group effect, *p *=* *.30
van Spijker et al. (2014), Netherlands	RCT	*N* = 236 Adults with mild to moderate suicidal ideation; Age *M* = 40.93 years, *SD* = 13.7; Female *N* = 156 (66%)	6 weeks	Internet Web site. Unguided self‐help intervention primarily based upon CBT principles, but also including elements of DBT, problem‐solving therapy, and mindfulness‐based cognitive therapy. Delivered across six modules over 6 weeks	Access to Web site providing information on suicide	BSS	A significant time by group effect whereby there was greater improvement in suicidal ideation in the treatment group (*M* change = 4.47, *SD *=* *8.72) compared to the control group (*M* change = 2.30, *SD *=* *6.57), *p *<* *.05
van Spijker et al. (2018), Australia	RCT	*N* = 418 Adults currently experiencing suicidal thoughts; Intervention group *N* = 207; Age *M* = 39.5 years, *SD* = 11.9; Female *N* = 160 (77%) Control group *N* = 211; Age *M* = 41.7 years, *SD* = 11.9; Female *N* = 163 (77%)	12 months	Internet Web site. Six online modules drawing on CBT and DBT principles, Delivered across 6 weeks	Online 6‐week “living” programme, focused on general health and well‐being	Suicidal Ideation subscale of the CSSRS	No significant (*p *=* *.23) difference in treatment groups for the severity of suicidal ideation at posttreatment, or at six and 12 months follow‐up
Wilks et al. (2018), USA	RCT	Adults with suicidal ideation, high emotion dysregulation and a history of heavy episodic drinking Intervention group *N* = 30; Age *M* = 38.0 years, *SD* = 11.3; Female *N* = 20 (67%) Control group *N* = 29; Age *M* = 37.4 years, *SD* = 10.1; Female *N* = 21 (72%)	4 months	Internet delivered. Internet‐delivered DBT skills training. Delivered over 8 weeks	Waiting list	BSS	No significant Time by group effect on suicidal ideation (*p *=* *.22)

BSS, Beck Scale of Suicidal Ideation; CBT, Cognitive Behavioral Therapy; CI, Confidence Intervals; DBT, Dialectical Behavior Therapy; NA, Not Applicable; NR, Not Reported; NSSI, Nonsuicidal Self‐Injury; RCT, Randomized Controlled Trial; SB, Self‐injurious Behavior; SITBI, Self‐Injurious Thoughts and Behaviors Interview; TEC, Evaluative Conditioning.

### Sample and Intervention Characteristics

The included trials comprised a total of *n *=* *2,016 adults and adolescents. The majority of the studies included adult samples (*k *=* *16). Seven interventions involved established psychological therapies including CBT, DBT, and individual emotion‐regulation therapy, delivered via an Internet Web site (Bjureberg et al., 2018; Eylem, 2019; Hetrick et al., 2017; Robinson et al., 2016; van Spijker, van Straten, & Kerkhof, 2014; van Spijker et al., 2018; Wilks et al., 2018). Nine used mobile phone applications to deliver a toolbox of support including coping skills and strategies (often derived from approaches such as CBT and DBT) as well as signposting or crisis support (Bush et al., 2015, 2017; Kennard et al., 2018; McManama O'Brien, LeCloux, Ross, Gironda, & Wharff, 2016; O'Toole, Arendt, & Pedersen, 2019; Pauwels et al., 2017; Rizvi, Hughes, & Thomas, 2016; Stallard, Porter, & Grist, 2018; Tighe et al., 2017). Three interventions involved supportive text messages (Berrouiguet, Gravey, Le Galudec, Alavi, & Walter, 2014; Chen, Mishara, & Liu, 2010; Kodama et al., 2016). One intervention involved the use of audio and text messages relating to coping skills and strategies, which followed on from face‐to‐face support (Marasinghe et al., 2012). Another intervention employed an aversive behavioral conditioning‐based intervention (Therapeutic Evaluative Conditioning; TEC), delivered via mobile application, whereby NSSI‐related stimuli were paired with aversive stimuli (Franklin et al., 2016). Lastly, one intervention involved Autobiographical Self‐Enhancement Training (ASET) whereby participants undertook a written (or typed) exercise that helped them identify and focus on positive personal attributes (Hooley, Fox, Wang, & Kwashie, 2018). Kennard et al. (2018) combined a mobile application with novel face‐to‐face therapy, and we therefore focus on outcomes linked specifically to application use.

### Potential for Bias

A summary of the potential for bias assessment is displayed in Table 2. Nine studies used single‐arm or pre–post designs and so were not rated for items related to sequence generation or allocation concealment since participants were not allocated to treatment groups. However, the results of such studies should be interpreted with caution since improvements cannot be attributed to the treatment itself, and may reflect other factors. There were a number of areas of recurring high potential for bias across studies. The potential for detection bias was high for 13 studies, where research staff undertaking assessments were not masked. Also, because it is usually not possible to mask participants to the fact they are receiving therapy, all included studies were rated high for performance bias except two that used active control interventions. Twelve studies were also judged at high potential for selective reporting bias, largely due to the lack of preregistration of trial protocols. However, the potential for attrition bias was generally low across studies. Other sources of bias were identified for eleven trials related to nonvalidated measurement tools or small sample size. In summary, the research appears at a preliminary stage and improvements in design (great use of RCT designs, use of allocation concealment, and masking), sample size, and preregistration would be beneficial.

**Table 2:** Results of Potential for Bias Assessment

Study	Random sequence generation	Allocation concealment	Reporting bias	Other bias	Performance bias	Detection bias	Attrition bias
Berrouiguet et al. (2014)	NA	NA	High	High	High	High	Low
Bjureberg et al. (2018)	NA	NA	Low	High	High	High	Low
Bush et al. (2015)	Unclear	Unclear	High	High	High	High	Low
Bush et al. (2017)	Low	Unclear	High	Low	High	High	Low
Chen et al. (2010)	NA	NA	High	High	High	High	Low
Eylem (2019)	Low	Low	Low	High	High	Low	Unclear
Franklin et al. (2016)	Low	Unclear	High	Low	Low	Low	High
Hetrick et al. (2016)	Low	Low	Low	Low	High	Low	High
Hooley et al. (2018)	Low	Low	High	Low	Low	Low	Low
Kennard et al. (2018)	Low	Unclear	Low	Low	High	Low	Low
Kodama et al. (2016)	NA	NA	High	High	High	High	Low
Marasinghe et al. (2012)	Unclear	Unclear	High	Low	High	Low	Low
McManama O'Brien et al. (2016)	NA	NA	High	High	High	High	Low
O'Toole et al. (2019)	Unclear	Low	High	Low	High	High	High
Pauwels et al. (2017)	NA	NA	High	High	High	High	High
Rizvi et al. (2016)	NA	NA	High	High	High	High	Low
Robinson et al. (2016)	NA	NA	Low	High	High	High	Low
Stallard et al. (2018)	NA	NA	Low	High	High	High	High
Tighe et al. (2017)	Low	Unclear	Low	Low	High	High	Low
van Spijker et al. (2014)	Low	Low	Low	Low	High	Low	Low
van Spijker et al. (2018)	Low	Low	Low	Low	High	Low	High
Wilks et al. (2018)	Low	Unclear	Low	Low	High	Low	Low

NA, not applicable.

### Self‐Injurious Behaviors Not Otherwise Specified

Two trials (Kodama et al., 2016; Stallard et al., 2018) investigated the effect of the therapeutic interventions on SB, where suicidal intent was not specified. In psychiatric outpatients, a supportive text messaging service was associated with a significant effect of time characterized by decline in the frequency of SB (from *N* = 8, 27.6%, to *N* = 2, 6.9%, *p *=* *.03) over 6 months. For young people with a history of STB using a mobile therapy app, the number who reported any recent SB declined from 78.8% to 66.7%, and of those who reported SB, 68.2% reported the frequency had reduced. However, the small scale of these trials (*n* = 30–44) and lack of a control group means these results remain preliminary.

### Nonsuicidal Self‐Injury

Four trials (Bjureberg et al., 2018; Franklin et al., 2016; Hooley et al., 2018; Rizvi et al., 2016) investigated the effect of therapeutic interventions on NSSI with mixed findings. Two trials (Bjureberg et al., 2018; Rizvi et al., 2016) reported significant effects of time characterized by reductions in NSSI for individuals using a DBT‐informed mobile app (frequency of app use associated with declines in NSSI over 3 months) or online emotion‐regulation therapy (e.g., 69% reduction in NSSI frequency over 6 months, *d *=* *1.36, 95% CI: 1.12, 1.63). However, the absence of a control group means these effects cannot be attributed to the interventions. Franklin et al. (2016) studied a behavioral conditioning‐based intervention delivered via a mobile phone application with mixed findings across three studies. In two of the three studies, those in the active treatment reported fewer NSSI episodes during the treatment month than those in the control condition (Incident Rate Ratio for treatment group = 0.72–0.88). However, none of these treatment effects were maintained during the follow‐up month. Hooley et al. (2018) found no evidence of a beneficial treatment effect on NSSI compared to active control conditions (*g *=* *−0.14, 0.07), though all groups experienced a decline in NSSI episode count.

### Suicide Attempt

Three RCTs investigating the effect of the therapeutic intervention on suicidal behavior did not identify any significant treatment effects, but power was likely adversely affected by the low base rate of such behavior (Hetrick et al., 2017; Hooley et al., 2018; van Spijker et al., 2018). van Spijker et al. (2018) reported small treatment effects following online therapy (*d *=* *−0.11, 0.15; calculated from marginal means). A trend toward reductions in suicidal behavior was, however, noted by Hetrick et al. (2017), with no suicide attempts at either 10 weeks or 22 weeks of follow‐up in the intervention group compared to three and two participants attempting suicide in the control group. A fourth study by Franklin et al. (2016) also reported fewer participants engaging in suicidal behavior in the active treatment group compared to the control group in two of their studies (study 1: 5 vs. 4 individuals; study 2: 4 vs. 8 individuals; study 3: 3 vs. 5 individuals). In two further studies, the use of a therapeutic mobile app was not associated with a decline in the risk of suicide attempts (Kennard et al., 2018; Rizvi et al., 2016).

### Suicidal Ideation

**Figure 2:**
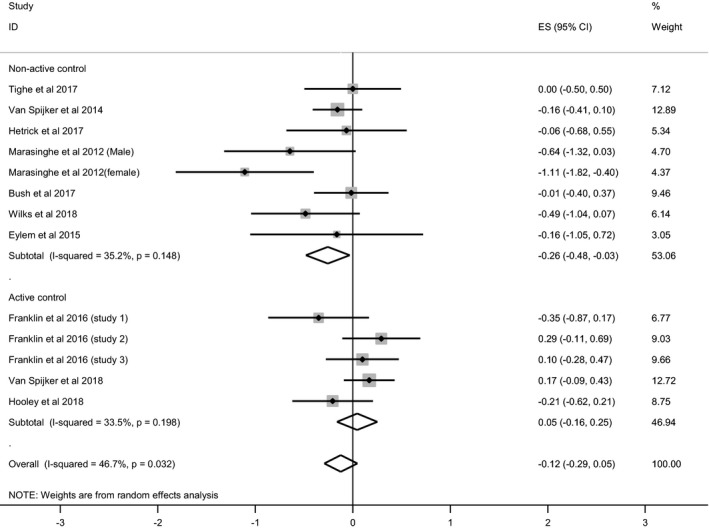
Forest plot of treatment effects on suicidal ideation, subdivided by control group (active or nonactive).

Eleven RCTs (14 samples) included suicidal ideation as an outcome. In one trial (Kennard et al., 2018), the effect of a mobile application could not be separated from a novel face‐to‐face intervention within the treatment group comparisons, so these data were not included in the meta‐analyses. Endpoints ranged from 4 weeks to 6 months. Only two trials reported significant treatment effects (differences between treatment arms; Marasinghe et al., 2012; van Spijker et al., 2014). A random‐effects meta‐analysis, focusing on the endpoint closest to posttreatment, indicated a nonsignificant effect of treatment upon suicidal ideation, *k *=* *13*, g *=* *−0.12 (95% CI: −0.29, 0.05), *I* ^2^ = 47%, with a moderate degree of inconsistency (see Figure 2). It is notable that treatment effects were often less favorable in those studies where control tasks or treatments other than TAU were used. When focusing only on trials with TAU as a comparator, there was a significant beneficial treatment effect, *k *=* *8, *g *=* *−0.26 (95% CI: −0.48, −0.03), *I* ^2^ = 35%, with moderate inconsistency apparent (see Figure 2). The meta‐analysis was also repeated with three trials (all with TAU as a comparator) where mean change data were available, *g *=* *−0.26 (95% CI: −0.48, −0.05), *I* ^2^ = 0%, leading to a significant effect of treatment on suicidal ideation. A final meta‐analysis focused on outcomes at 3–6 months of follow‐up (*k *=* *5), but did not identify a beneficial effect on suicidal ideation, *g *=* *−0.18 (95% CI: −0.49, 0.12), *I* ^2^ = 37%.

The results emerging from single‐arm studies of mobile‐ or Internet‐based intervention were largely favorable. Four single‐arm studies all reported a decline in suicidal ideation following the intervention. This was apparent for CBT‐based online intervention aimed at students with recent suicidal ideation (*d *=* *0.66; Robinson et al., 2016), text messaging interventions aimed at psychiatric outpatients (Kodama et al., 2016), and a DBT‐based mobile intervention aimed at adults diagnosed with borderline personality disorder (30% of sessions with the app were associated with a decline in urges to SB; Rizvi et al., 2016). Kennard et al. (2018) did not find a significant relationship between app use and changes in ideation. A further mobile phone app for adults with suicidal ideation did not lead to a reduction in ideation, though the study was likely under‐powered (*N* = 21) given the 1‐week follow‐up period (Pauwels et al., 2017).

### Acceptability

Self‐reported acceptability was good where assessed (*k *=* *13). For text message‐based services, participants in three studies found the interventions to be helpful (80%–93%), and a good way to stay in touch with services (93%; Berrouiguet et al., 2014; Chen et al., 2010; Kodama et al., 2016). However, in one of these studies participants were more divided around whether the service benefited their psychological health, and suicidal thoughts or behavior (40%–60% felt the service was helpful in these areas; Berrouiguet et al., 2014). For mobile phone apps, participants typically found these helpful or were satisfied with the content (e.g., 84%–89%; Bush et al., 2015, 2017; McManama O'Brien et al., 2016), and would consider using the apps again (e.g., 83%–94%; Bush et al., 2015, 2017; Rizvi et al., 2016; Stallard et al., 2018). In one study, helpfulness ratings were more ambivalent (*M* rating = 2.46 of 5) but higher when participants were specifically asked if the app would be helpful for SB (*M* rating = 3.23 of 5; Rizvi et al., 2016). In two other studies, the majority of participants also felt the apps would help with STB (60%–80%; Pauwels et al., 2017; Stallard et al., 2018).

Internet‐delivered CBT and DBT approaches were largely seen as helpful (91% of participants; Robinson et al., 2015) or rated useful across two studies (*M* usefulness rating = 3.91 of 5; Wilks et al., 2018). Overall satisfaction was only moderate for one Internet‐delivered emotion regulation therapy (*M* rating = 17.8 of 32; Bjureberg et al., 2018). Hooley et al. (2018) also reported relatively low acceptability ratings for their online therapeutic writing task, with low ratings for how much participants understood the task and planned to continue using it (average scores below 3, indicative of disagreement).

### Feasibility

With regard to engagement rates, most interventions reported good rates of initial engagement but often rates of completion, or continued use of the intervention were more limited (reported by *k *=* *11). Hetrick et al. (2016) reported that, on average, participants commenced five of eight modules, with only eight of the 26 participants completing all eight modules. Rates of initial engagement with therapy Web sites were high (88%–92% starting the intervention; van Spijker et al., 2018, 2014) but fewer accessed at least three of the six modules (44%–56%). Wilks et al. (2018) identified a declining trend in engagement, but note that technical problems may have been a contributing factor. Similarly for mobile phone apps while good rates of initial engagement were noted by three studies (e.g., 71%–83% using the app at least once; Franklin et al., 2016; Kennard et al., 2018; O'Toole et al., 2019), a trend toward a decline in use was also identified in one (Franklin et al., 2016). For example, Bush et al. (2015) report that 88% using app over 2 weeks versus 59% over 6 weeks. Completion rates for other mobile apps (85%; Tighe et al., 2017) and Web sites (average completion rate of 9.7 of 11 modules; Bjureberg et al., 2018) were good. Hooley et al. (2018) also reported good engagement (mean completion of 21.31 sessions of a possible 28), but engagement was incentivized. Importantly, while a decline in usage might indicate problems with feasibility, this may also reflect other processes such as participants no longer needing the support of the intervention due to recovery.

## Discussion

The aim of this review was to evaluate the efficacy and acceptability of mobile‐ and Internet‐based interventions for STB. Twenty‐two studies covering mobile phone applications, text‐based services, and Web site‐based interventions with a focus on STB prevention were identified. The current review extends the literature by focusing specifically on interventions where a primary aim is STB prevention (as opposed to interventions primarily directed at related difficulties like depression). This is important since intervention effects on STB prevention may be better when this is the primary focus (Christensen et al., 2014; Tarrier et al., 2008). The majority of identified studies (*k *=* *17) were not included in the last review on this topic (Witt et al., 2017). Results suggest that evidence for the efficacy of Internet‐ and mobile‐based interventions for STB remains limited. Evidence of possible reductions in SB largely derives from single‐arm, noncontrolled studies. Evidence of a beneficial effect on suicidal ideation was apparent compared with treatment as usual but not when active controls were also considered. However, two of three trials with active control conditions featured more atypical interventions (Franklin et al., 2016; Hooley et al., 2018), so this may have also explained the differing results. Findings were mixed regarding NSSI, but there was preliminary support for DBT‐based applications. Evidence for more focused behavioral or autobiographical interventions (Franklin et al., 2016; Hooley et al., 2018) with regard to NSSI and suicidal ideation is currently lacking. There is currently no clear indication that therapies based on a particular modality (e.g., text messages, Internet, or mobile apps) are more effective.

This review further builds on previous work by synthesizing the data concerning acceptability and feasibility. The acceptability of interventions was largely supported. Questions tended to center broadly on how accessible and helpful interventions appeared to participants. More in‐depth exploration of issues that might be particular to Internet‐ or mobile‐based intervention, such as the ability to develop a therapeutic alliance or feeling facilitated in better understanding one's SB risk and engaging in problem solving around this, would be beneficial. Regarding feasibility, there was evidence that engagement or usage of interventions may decline with time, but this may be due to several reasons and further examination of this is warranted. Technological problems and issues with the usability of apps or Web sites (e.g., nonintuitive interfaces) represent a further factor that may affect acceptability and feasibility. Distinguishing between these issues is likely to be difficult in practice. Careful initial pilot testing of software for technical issues prior to fuller evaluation is likely to be beneficial. Moreover, the use of more detailed qualitative interviews may help to disentangle issues with usability of the technology from issues related to the acceptability or feasibility of the intervention content.

While there is evidence regarding the potential benefits of Internet‐ and mobile‐based interventions for mental health difficulties (Firth et al., 2017; Josephine et al., 2017; Richards et al., 2015; Stratton et al., 2017), the benefits for interventions focused specifically on STB prevention are less established. The majority of identified studies concerned CBT‐ and DBT‐informed interventions (both Internet and mobile application based), and there is preliminary evidence that these are acceptable and can be potentially helpful in reducing suicidal thinking. An important next step is to further develop this evidence base by undertaking larger‐scale RCTs of these existing interventions. These RCTs should consider outcomes related to SB (NSSI and suicide attempts) in addition to suicidal ideation. As the majority of identified studies concerned adults, further trials of interventions aimed at adolescents and young people at risk of STB would also be beneficial.

A broader issue relates to how such interventions compare to face‐to‐face psychological interventions, and whether there are any gains or losses in efficacy. None of the included trials compared interventions to active face‐to‐face counterparts. However, noninferiority trials for problems such as depression and posttraumatic stress disorder suggest similar treatment effects are possible (Acierno et al., 2017; Ly et al., 2015). Further noninferiority trials would be beneficial, but these are difficult to conduct as expected differences might be small and result in large sample size requirements. It is important to explore whether Internet‐ or mobile‐based interventions work best as an adjunct to face‐to‐face intervention, or can be beneficial as stand‐alone interventions. We would hypothesize that Internet‐ or mobile‐mediated interventions for SB work best when they facilitate a working alliance with a real‐world therapist, but overcome barriers to access (e.g., stigma, distance, cost); however, further research is needed to test this possibility. SB is a highly heterogeneous problem, which can have a wider range of underlying functions and triggers (Hawton et al., 2012; Taylor et al., 2018). This may pose further challenges to Internet‐ or mobile‐based interventions if they are not able to tailor their approach to the needs of a particular client. However, developments in machine learning raise the possibility of more “intelligent” therapy application (Kelly et al., 2012). Machine learning could involve software using data inputted by the user to “learn” over time the optimal times or situations to introduce particular interventions. This would mean that the treatment package or options that individuals receive could be personally tailored to their particular difficulties, potentially leading to more effective interventions. It remains to be established whether machine learning could approximate the idiosyncratic formulation‐driven treatment that can be offered within face‐to‐face talking therapies (e.g., Tarrier & Johnson, 2016).

The current review was limited by excluding non‐English language papers, which may have meant otherwise relevant research was not included. Similarly, the small number of included studies prevented us from applying approaches such as subgroup analysis or meta‐regression to better understand the possible causes of heterogeneity in effects. We combined effects from several different types of intervention (mobile apps, therapy Web site, text message service), and this is likely one factor contributing to heterogeneity in meta‐analyses. Likewise, the small number of studies precluded tests of publication bias, which may be a factor biasing findings.

Internet‐ and mobile‐based interventions have the potential to help individuals at risk of STB, but further trials are needed to confirm their efficacy. When considering Internet‐ and mobile‐based interventions together, a larger number of RCTs have been undertaken concerning problems like depression and anxiety (Firth et al., 2017; Josephine et al., 2017; Richards et al., 2015; Stratton et al., 2017). The comparative smaller number of RCTs that focus on STB may be the result of the often greater risk and complexity associated with STB. The results of this review indicate that it is possible to develop mobile‐ and Internet‐based interventions for STB that are acceptable and potentially helpful in reducing suicidal ideation. We encourage future trials to focus on existing interventions that have already shown promise but require further evaluation in larger samples using controlled designs. In developing and evaluating Internet‐ and mobile‐based interventions, greater interdisciplinary collaboration would be valuable. For example, collaboration between computer scientists, software engineers, clinicians, and mental health researchers may help improve the acceptability and usability of such interventions, and help support the development of interventions drawing on machine learning principles. Likewise, involving those with lived experience of STB in intervention development is important in ensuring interventions feel acceptable and useful to the individuals they have been designed to help.

## Supporting information


**Appendix S1.** Departures from protocol.
**Appendix S2.** Original Search strategy.
