# Supplementary material for: A Systematic Review of the Evidence Supporting Mobile‐ and Internet‐Based Psychological Interventions For Self‐Harm
Source: Suicide Life Threat Behav. 2019 Aug 26;50(1):151–79. doi: 10.1111/sltb.12583 (PMC7027458; doi:10.1111/sltb.12583)
Supplement: Supplementary file 1 — Appendix S1. Departures from protocol. Appendix S2. Original Search strategy. [file SLTB-50-151-s001.docx]

**Appendix S1**

**Departures from protocol**

- For consistency with US terminology we replaced the term “self-harm” with Self-Injurious Thoughts and Behaviour (STB).
- The definition of acceptability was adjusted and feasibility was included as a further outcome.
- In response to comments from reviewers, the search strategy was adjusted to make it more comprehensive and inclusive. This included the addition of further searches of Association of Computing Machinery Digital Library. The original search strategy is detailed in Appendix S2.

**Appendix S2**

**Original Search strategy**

Electronic databases (PsycINFO, Web of Science, and MEDLINE) were searched from earliest date till Janary 2019 using the following subject terms and Boolean operators: (“mobile health” OR “text message” OR “E-health” OR m-health OR “mobile intervention” OR “mobile-based intervention” OR “mobile apps” OR “apps” OR computerised OR computerized OR “online self-help” OR “online intervention” OR “online therapy” OR “web therapy” OR “web intervention” OR ICT) AND (“self-harm” OR “self-injury” OR DSH OR NSSI OR “self-burn*” OR “self-mutilation” OR “self-cutting” OR suicid*). The asterisks indicate wildcard operators. Database specific subject headings were also used where these map onto search terms. These additional terms are displayed in Supplementary Table 1, below.

The data screening was done in two steps. Firstly, titles and abstracts were screened by two reviewers independently. Abstracts not fulfilling the inclusion criteria were excluded. Where it was uncertain if studies met inclusion criteria, they were retained for the next stage of screening. Secondly, full text articles were screened out on the basis of inclusion/exclusion criteria. Both stages of screening were completed in parallel by two independent reviewers (active researchers with master’s level qualifications in psychology) and discrepancies were discussed and resolved by a third reviewer (doctorate in psychology and extensive experience of systematic reviews).

Supplementary Table 1

Database Specific Subject Headings Used in Literature Searches.

| Database | Subject Headings |
| --- | --- |
| **PsycInfo** | cellular phones, mobile devices, information technology, telemedicine, computer applications, computer assisted therapy, online therapy  self-mutilation; suicidal ideation; self-destructive behaviour; attempted suicide; self-injurious behaviour; suicide |
| **Medline** | telemedicine; test messaging; mobile applications; therapy, computer assisted; |
|  | self-mutilation; suicide, attempted; suicide; self-injurious behaviour |
